# An approach for measuring the spatial orientations of a computed‐tomography simulation system

**DOI:** 10.1120/jacmp.v15i2.4544

**Published:** 2014-03-06

**Authors:** Meng Chia Wu, Ramani Ramaseshan

**Affiliations:** ^1^ Department of Radiation Oncology BC Cancer Agency — Abbotsford Centre Abbotsford BC Canada

**Keywords:** spatial orientation, angular deviation, computed‐tomography (CT) simulator

## Abstract

The quality assurance tests for measuring the spatial orientations between tabletop, external patient positioning lasers, couch longitudinal moving direction, and imaging plane in a CT simulation system are a complicated and time‐consuming process. We proposed a simple and efficient approach to acquire the angular deviations of spatial orientations between these components. An in‐house cross‐jig was used in this study. We found a relationship between the orientations of the jig's arms shown on the CT images and the orientations of the components in a CT simulator. We verified this relationship with 16 misalignment orientations of known errors, to simulate all possible deviation situations. Generally, the tabletop and external lasers system are mounted separately in a CT simulation system; the former is on the couch trail, the later is on the wall and ceiling. They are independent to each other and will cause different effects on CT images. We only need two scans to acquire the angular deviations of our system: i) when aligning the cross‐jig with tabletop, we can check the orientations between the tabletop, couch longitudinal moving direction, and imaging plane; ii) while aligning the cross‐jig with the external axial lasers, we will know the angular deviation between the lasers, couch longitudinal moving direction, and imaging plane. The CT simulator had been carefully examined by performing the QA procedures recommended by the AAPM Task Group 66. The measurements of the spatial orientations using the proposed method agree well with TG 66 recommendations. However, the time taken to perform the QA using our method is considerably shorter than the method described in TG 66 — 5 minutes versus 30 minutes. The deliberate misalignment orientations tests with known errors were detected successfully by our in‐house analysis program. The maximum difference between the known errors and the measured angles is only 0.07°. We determined that the relationship between the orientations of the jig's arms and the orientations of the CT components. By means of quantifying the deviations in degree we can correct the errors accurately. This approach can also be used to inspect the spatial orientations of other imaging systems, such as PET‐CT and MRI.

PACS number: 87.57.Q‐

## I. INTRODUCTION

Computed‐tomography (CT) scanner has been used for a few decades. It has become the major imaging tool in radiation oncology facilities,^1^, ^2^, ^3^ due to the fact that a CT scanner can provide high image resolution and the CT numbers for different materials which, in turn, can provide radiation attenuation information. The CT scanner which allowed acquiring volumetric CT images of patient and performing virtual simulation process in radiotherapy department is called a CT simulator.^4^, ^5^ Due to the requirements of patient positioning and immobilization for radiation treatment, a CT simulator is typically equipped with a flat tabletop for simulating the flat treatment couch and a set of external patient positioning lasers for patient alignment.^6^, ^7^, ^8^

**Figure 1:**
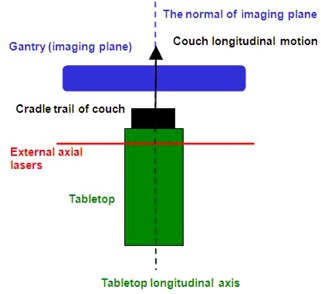
A pictorial graph which shows the spatial orientations in a CT simulation system. The angle between the couch longitudinal moving direction and the normal of imaging plane should be 0°. The angle between the longitudinal axis of tabletop and the couch longitudinal moving direction should be 0°. The angle between the external axial lasers and the imaging plane should also be 0°.

The flat tabletop is usually mounted on a cradle‐shaped couch trail of a CT scanner and is often indexed to accommodate registration of patient immobilization devices. The cradle trail will move back and forth along with the tabletop, while acquiring a CT scan. It is expected that the longitudinal axis of the tabletop is parallel with the couch longitudinal moving direction and is orthogonal with the imaging plane. The external patient positioning lasers in a CT simulation system are used to tattoo and place positioning markers on patient's skin. These markers in the patient CT images are used in treatment plans, while the tattoos are used to reproduce the patient's position in the radiation treatment room. Hence, the lasers in CT simulator should have the same accuracy and spatial orientation requirement as the lasers in radiation treatment room. Generally the external lasers in a CT simulator system consist of three separate portions: two side‐wall lasers identify the coronal and axial planes, and one ceiling laser is used to identify the sagittal and axial planes. According to the patient positioning requirement, the axial plane defined by the external lasers is in a fixed distance from the imaging plane and is parallel with the imaging plane. In addition, this axial plane is orthogonal with the couch longitudinal moving direction. In the following discussion, we focus on the orientation of the axial plane, which is defined by the external lasers. A pictorial graph is showed in Fig.1 to represent the correct spatial orientations in a CT simulation system.

The spatial orientations of tabletop and external lasers with respect to the couch longitudinal moving direction and imaging plane must be precise. If this is not the case, the patient's position may not be reproduced in the treatment room and the patient's position in the volumetric images may not be accurate.^9^, ^10^, ^11^, ^12^ Potentially, this could result in geometric missing treatment on the lesion and/or a severe damage on the critical organs. The important point to note is the tabletop and external lasers are usually attached to a CT scanner and may not be integrated well with the scanner. Therefore, an independent quality assurance (QA) test is necessary to make sure these components are integrated with the CT scanner very well. Several studies have reported the importance and method to perform the orientation QA.^10^, ^13^, ^14^, ^15^, ^16^ However, the recommended tests to find out the spatial orientations between tabletop, external lasers, couch movement, and imaging plane involve several CT scans and multiple phantom arrangements. It is a quite complicated and time‐consuming process. Furthermore, most of the tests are measuring the difference of linear distances between CT markers in slices, rather than determining the exact angle between components. The issue to be aware of is, those errors are the measurements in length. It is insufficient and could be easily misunderstood for using a linear difference to describe the deviation of an angular orientation.

The present study describes a simple and efficient method to acquire the spatial orientations between tabletop, external lasers, couch longitudinal moving direction, and imaging plane. This approach allows analyzing the coordinates of CT markers to obtain the angular deviations between these four components in a short time from two CT scans.

## II. MATERIALS AND METHODS

**Figure 2:**
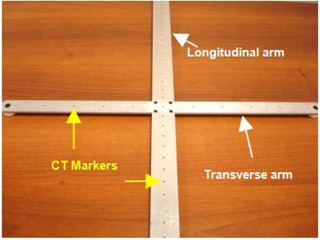
The longitudinal arm of the jig is orthogonal with the transverse arm and there are several CT markers inlaid in the arms: 11 markers on the transverse arm and 23 markers on the longitudinal arm for other tests. In this study, we only need five CT markers for the orientation analysis. There are two plugs at the ends of the transverse arm which can clamp to the notches on the sides of tabletop.

An in‐house cross‐jig, as shown in Fig. 2, was used in our study. The cross‐jig was constructed in such a way that the longitudinal arm of the jig is perpendicular to the transverse arm (i.e., the angle between longitudinal arm and transverse arm is 90°). The arms are made by aluminum. Several CT markers of diameter of 1.5 mm were embedded in both arms: 11 markers in the transverse arm are used mainly for verifying the image scaling, while 23 markers in the longitudinal arm are used for measuring the couch moving distance. The CT markers were visible on CT images without much artifact. Five CT markers were used for the orientation analysis. A central marker was placed at the centre of jig and the remaining four markers are at the ends of the longitudinal and transverse arm and 200 mm away from the central marker, respectively. These CT markers are used to represent the positions of both arms on CT images and the orientations of the arms in the volumetric images can be identified. There are two plugs at the ends of the transverse arm which served as exact couch attachment, and hence the jig could be positioned accurately and reproducibly on the tabletop. Care was taken to manufacture the jig and, as well, was verified such that the longitudinal arm was parallel to the longitudinal axis of the tabletop when the jig is locked on to the tabletop.

**Figure 3:**
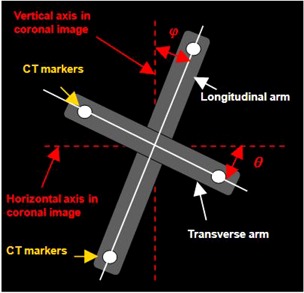
A pictorial representation shows the definition of ϕ and θ from the coronal view of volumetric jig CT scan. ϕ is the acute angle formed by the longitudinal arm of the jig and the longitudinal axis of the image, and θ is the acute angle formed by the transverse arm of the jig and the transverse axis of the image. The angel will be positive if the arm is in clockwise direction with respect to the axis; otherwise, it will be negative.

In a volumetric CT image set, we define the vertical axis of the coronal image is the axis coincides with the patient's superior‐inferior direction for a patient oriented head‐first supine into the gantry and the horizontal axis coincides with the patient's left‐right direction. While we clamp the cross‐jig on the tabletop and perform a scan, in the scenario of ideal spatial orientations in CT simulator, the coronal view of reconstructed jig images will show the arms are aligned with the image axes. That is, the longitudinal arm is parallel with the vertical axis of the image and the transverse arm is parallel with the horizontal axis of the image. If the spatial orientations in the CT simulator are not ideal, the coronal view will show the arms to project a small angle with respect to the image axes, as shown in Fig. 3. We defined ϕ as the acute angle formed by the longitudinal arm of the jig to the vertical axis of the image, and θ as the acute angle formed by the transverse arm of the jig to the horizontal axis of the image. We also defined that the angle would be positive if the arm was in clockwise direction with respect to the axis, and it would be negative in the other direction. In the subsequent discussion, we will demonstrate that ϕ and θ may not be the same and will depend on the spatial orientations between tabletop, external lasers, couch longitudinal moving direction, and imaging plane.

Generally, the tabletop and external lasers are mounted separately. The error of tabletop orientation will not affect the positioning of the external lasers. The relationships between the spatial orientations in a CT simulator and ϕ and θ are described below.

### A. The tabletop orientation

**Figure 4:**
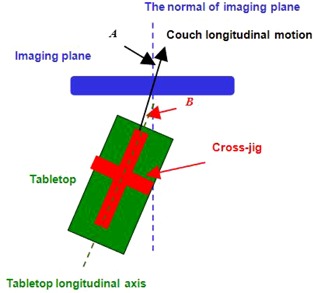
This is the bird's eye view of a CT simulator when we clamp the jig on the tabletop. The angle A is defined as the acute angle formed by the couch longitudinal moving direction and the normal of the imaging plane, and angle B is formed by the longitudinal arm of the jig and the couch longitudinal moving direction.

In the situation that the spatial orientations between tabletop, couch longitudinal moving direction and imaging plane are not aligned (i.e., the longitudinal axis of tabletop is not parallel with the couch longitudinal moving direction and/or the couch longitudinal moving direction is not orthogonal with the imaging plane). When we attach the jig on the tabletop there will be an acute angle, defined as B, formed by the longitudinal arm of the jig and the couch longitudinal moving direction. B will be positive if the tabletop longitudinal axis is in clockwise direction with respect to the couch longitudinal moving direction; otherwise B will be negative. We also defined angle A as the acute angle formed by the couch longitudinal moving direction and the normal of the imaging plane. Similarly, A will be positive if the couch longitudinal moving direction is in clockwise direction with respect to the imaging plane; otherwise A will be negative, as shown in Fig.4.

**Figure 5:**
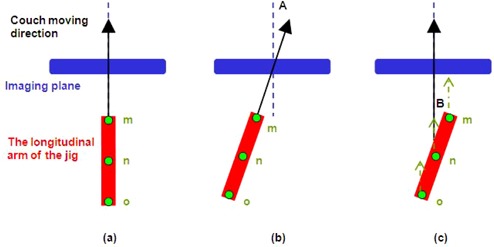
The pictorial representations show the derivation of the relationship between ϕ and A, B: (a) when A=0,B=0; (b) shows A≠0,B=0; (c) the situation of A=0,B≠0.

In order to derive equations for ϕ,θ, and A, B, let's first consider only the longitudinal arm of the jig. It can be seen in Fig. 5 that the bold red line represents the longitudinal arm (the longitudinal axis of the tabletop), the arrow represents the couch longitudinal moving direction, and the three points, m, n, o, on the bold red line represent the CT markers at the center and the ends. Figure 5(a) shows that when A=0,B=0 (i.e., everything is aligned perfectly), the CT scan will show point m, n, o with the same x, y coordinates on all image slices containing these points. In the reconstructed coronal view, the longitudinal arm of the jig will be parallel with the vertical axis of the image (i.e., ϕ=0). Figure 5(b) shows the instance when A≠0,B=0.

In such situation, the points m, n, o pass through the same position at the imaging plane during CT scan and will have same x, y coordinates, while the ϕ will still be 0 in the reconstructed coronal view. In the situation as shown in Fig. 5(c) where A=0,B≠0, points m, n, o pass through different positions at the imaging plane. The x, y coordinate positions between points m, n, o will be related to angle B. In the reconstructed coronal view, ϕ=B.

**Figure 6:**
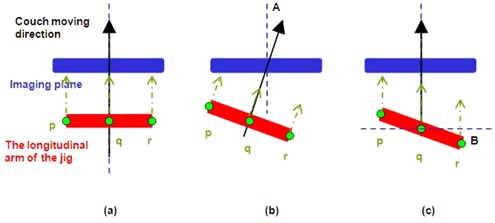
The pictorial representations show the derivation of the relationship between θ and A, B: (a) when A=0,B=0; (b) when A≠0,B=0; (c) shows the situation of A=0,B≠0.

Next, we consider only the transverse arm of the jig. Figure 6 shows the bold red line represents the transverse arm and three points, p, q, r, on the bold red line represent the CT markers at the center and the ends. When A=0,B=0 (Fig. 6(a)), the points p, q, r pass through the imaging plane at the same time. This will be so because the transverse arm of the jig is parallel with the horizontal axis of the image (i.e., θ=0) in the reconstructed coronal view. In the condition where A≠0,B=0 (Fig. 6(b)), the points p, q, r pass through the imaging plane at different time during CT scan. The x, y coordinate positions of points p, q, r will be related to angle A. Hence in the reconstructed coronal view, θ=A. In Figure 6(c), when A=0,B≠0, points p, q, r also pass through the imaging plane at different time. The x, y coordinate positions of points p, q, r will be related to angle B. In the reconstructed coronal view, θ=B. Combining the outcomes mentioned above we derive the following equation:

(1) $$\begin{array}{l} \phi =B\\ \theta =B+A \end{array}$$

Therefore, by obtaining the coordinates of CT markers from CT images, we are able to calculate the angular deviation between the tabletop, couch longitudinal moving direction, and imaging plane.

### B. The external axial lasers orientation

**Figure 7:**
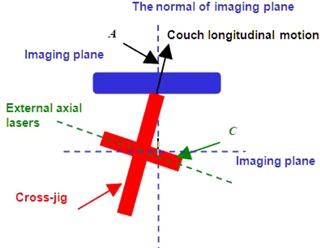
The bird's eye view of a CT simulator with the transverse arm of the jig aligned with the external axial lasers.

The discussion is similar to the one we mentioned above, where the spatial orientations between the external axial lasers, couch longitudinal moving direction, and imaging plane are not aligned. When the transverse arm of the jig is aligned to the external axial lasers, there will be an acute angle, defined as C, formed by the transverse arm of the jig and the imaging plane. If the external axial lasers are misaligned in clockwise direction with respect to the imaging plane, C will be positive; otherwise C will be negative (see Fig. 7). The definition of angle A is the same as mentioned above.

Following the interpretation of tabletop orientation, we can derive the equations of ϕ,θ, and A, C:

(2) $$\begin{array}{l} \phi =C‐A\\ \theta =C \end{array}$$

Similarly by obtaining the coordinates of CT markers from CT images, we will be able to calculate the angular deviation between the external axial lasers, couch longitudinal moving direction, and imaging plane.

**Figure 8:**
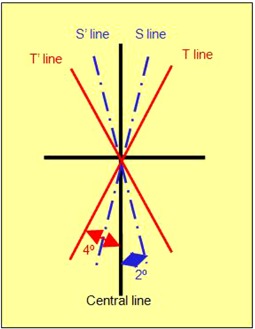
A central line on a paper corresponds with the longitudinal central axis of the tabletop. Four lines were generated on the paper: two were angled 2° in clockwise and counterclockwise direction with respect to the central line, called S and S', respectively, and the other two lines were angled 4° in each direction with respect to the central line, called T and T', respectively.

In order to verify the equations mentioned above, we designed 16 misalignment orientation tests with known errors by printing them on a paper, as shown in Fig. 8. The paper was placed accurately on the tabletop where the central line of the paper was aligned to the longitudinal axis of the tabletop. Four lines were generated, out of which two of them were angled 2° clockwise and counterclockwise to the central line, represented by S and S', respectively. The other two lines were angled 4° in clockwise and counterclockwise to the central line, representing T and T'.

The 16 possible misalignments comprise eight due to misalignment between tabletop, couch longitudinal moving direction, and imaging plane. The other eight possible scenarios are due to misalignments between orientation of external axial lasers, couch longitudinal moving direction, and imaging planes. To verify Eq. set (1), the details of the eight possible misalignments between tabletop, couch longitudinal moving direction, and imaging plane are described below. A CT scan was performed for each of the eight possible misalignments and the errors were calculated respectively.

Step 1. To simulate that the tabletop has 2° angular deviation clockwise with respect to the couch longitudinal moving direction but the couch longitudinal moving direction is orthogonal with the imaging plane, we aligned the longitudinal arm of the jig with S line to represent the tabletop orientation and performed a regular axial scan (i.e., the couch moves along the central line on the paper while scanning). Then, we did the same scan when the longitudinal arm of the jig aligned with s' line to simulate the deviation in the opposite direction.

Step 2. To simulate that the longitudinal axis of tabletop is parallel with the couch longitudinal moving direction but the couch longitudinal moving direction has 2° angular deviation clockwise with respect to the normal of imaging plane, we aligned the longitudinal arm of the jig with S line to represent the tabletop orientation. After acquiring each axial image, we manually moved the jig along S line with 1.25 mm steps until the whole jig was scanned. Then, we did the same scan when aligning the longitudinal arm of the jig with s' line and moving the jig along s' line to simulate the deviation in the opposite direction.

Step 3. To simulate that the couch longitudinal moving direction has 2° angular deviation clockwise with respect to the normal of imaging plane and the tabletop has 2° angular deviation clockwise with respect to the couch longitudinal moving direction, we aligned the longitudinal arm of the jig with T line. After acquiring each axial image, we manually moved the jig along S line with 1.25 mm steps until the whole jig was scanned (the longitudinal arm of the jig still aligned with T line). Then, we did the same scan when aligning the longitudinal arm of the jig with the central line on the paper and moving the jig along S line to simulate the deviation in the opposite direction.

Step 4. Similar to Step 3, but the couch longitudinal moving direction has 2° angular deviation in counterclockwise direction with respect to the normal of imaging plane and the tabletop has 2° angular deviation in clockwise and counterclockwise direction with respect to the couch longitudinal moving direction. We aligned the longitudinal arm of the jig with the central line on the paper and T' line, respectively, and after acquiring each axial image, we manually moved the jig along s' line with 1.25 mm steps until the whole jig was scanned.

The similar procedures have also been performed to create the orientation errors between the external axial lasers, couch longitudinal moving direction, and imaging plane for verifying Eq. set (2). The only difference is that the transverse arm of the jig now is representative of the external axial lasers.

For this study, the GE LightSpeed RT16 CT simulator was used (GE Healthcare, Waukesha, WI). Scans were performed using 100 kVp, 250 mAs, 50 cm field of view, and 1.25 mm slice thickness of axial scan for all images. We developed analysis program in MATLAB 7 (The MathWorks, Natick, MA). The analysis program can display DICOM images and automatically detect the coordinates of CT markers in three dimensional space based on a preset Hounsfield unit number. The center of CT marker is determined by taking into account the coordinates of each marker on different slices, and weights it to its intensity of each pixel in the CT images. Using Eq. sets (1) and (2), the software can quickly calculate the angular deviations of spatial orientations in the system.

## III. RESULTS

In order to compare, we used the same cross‐jig and carefully examined the spatial orientations of our system by performing the QA procedures recommended by the AAPM Task Group 66 (TG 66).^(16)^ The orientation errors of the CT simulator are all within 0.75 mm by visual estimation. We also used a MATLAB program to determine the coordinates of CT markers and calculate the angular deviations from the images based on TG 66 QA protocol. We call these angular deviations “inherent deviations”. Next, we performed the proposed approach to acquire the angular deviations of the system. The comparison of both results is listed in Table 1. We also counted the time spent on each method: for TG 66, we needed about 30 minutes to perform all deviation tests; with our new approach, it only took 5 minutes. The effects from couch sag and image distortion were determined to be very small for our system, since the angle of couch sag we measured was less than 0.1° and the image distortion was less than 0.2 mm error throughout a 40 by 40 cm field‐of‐view range.

As for the 16 misalignment simulation tests, the first eight are related to the tabletop orientation and the other eight are connected with the external axial lasers orientation; the results are described below, respectively.

**Table 1:** The comparison of QA results from the tests of TG 66 and the proposed method. The tests were performed three times with both methods, respectively

	*Result of TG 66*	*Result of New M*
The angle between couch longitudinal moving direction and the normal of imaging plane (A)	$(1)+0.05^{\circ }$	$(1)+0.03^{\circ }$
	$(2)+0.06^{\circ }$	$(2)+0.04^{\circ }$
	$(3)+0.03^{\circ }$	$(3)+0.03^{\circ }$
The angle between tabletop longitudinal axis and couch longitudinal moving direction (B)	$(1)-0.02^{\circ }$	$(1)-0.02^{\circ }$
	$(2)-0.05^{\circ }$	$(2)-0.02^{\circ }$
	$(3)-0.07^{\circ }$	$(3)-0.04^{\circ }$
The angle between the plane defined by external axial lasers and imaging plane (C)	$(1)-0.09^{\circ }$	$(1)-0.01^{\circ }$
	$(2)-0.09^{\circ }$	$(2)-0.02^{\circ }$
	$(3)-0.08^{\circ }$	$(3)-0.02^{\circ }$

### A. Spatial orientations between tabletop, couch longitudinal moving direction, and imaging plane

**Figure 9:**
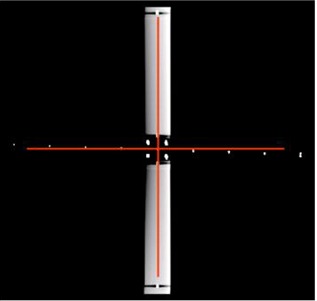
Coronal view acquired when the longitudinal arm of the jig was aligned with S line and the jig was manually moved along S line with 1.25 mm steps after acquiring each axial image. The red cross lines indicate the vertical and horizontal axes of the image. The white dots close to the horizontal axis are the CT markers on the transverse arm.

The reconstructed coronal image from one of the tabletop orientation simulations is shown in Fig. 9. This image was acquired while we aligned the longitudinal arm of the jig with S line and manually moved the jig along S line with 1.25 mm steps after acquiring each axial image. It is based on the simulation that there is a 0° angle between the longitudinal axis of tabletop and the couch longitudinal moving direction, and a +2° angle (clockwise) between the couch longitudinal moving direction and the normal of imaging plane. The transverse arm seems disappeared because we had to adjust the window/level of the CT image to see the markers more clearly. This distorted image shows that the transverse arm is not perpendicular to the longitudinal arm anymore. The angular deviations were calculated by our analysis program and the results are shown in Table 2. We also have performed the simulations with 1° orientation deviation. These simulations were performed three times and the results are listed in Table 3. These results have been subtracted by the inherent deviations measured by TG 66.

**Table 2:** The orientation deviations from the image shown on Fig. 6 were calculated by the MATLAB program. It is based on the situation that there is a 0° angle between the longitudinal axis of tabletop and the couch longitudinal moving direction and a $+2^{\circ }$ angle (clockwise) between the couch longitudinal moving direction and the normal of imaging plane

	*Expected Deviation*	*Measured Deviation*
The angle between couch longitudinal moving direction and the normal of imaging plane	$+2.00^{\circ }$	$+2.06^{\circ }$
The angle between tabletop longitudinal axis and couch longitudinal moving direction	$0.00^{\circ }$	$-0.03^{\circ }$

**Table 3:** The three measurements on the orientation simulations from −2° to +2° angle between tabletop, external axial lasers, couch longitudinal movement, and imaging plane

*The Expected Angular Deviation*	$-1.00^{\circ }$	$+1.00^{\circ }$	$-2.00^{\circ }$	$+2.00^{\circ }$
The measured angle between couch longitudinal moving direction and the normal of imaging plane (A)	$(1)-1.02^{\circ }$	$(1)+1.04^{\circ }$	$(1)-1.97^{\circ }$	$(1)+1.89^{\circ }$
	$(2)-1.05^{\circ }$	$(2)+1.02^{\circ }$	$(2)-1.91^{\circ }$	$(2)+1.92^{\circ }$
	$(3)-1.07^{\circ }$	$(3)+0.99^{\circ }$	$(3)-2.05^{\circ }$	$(3)+2.01^{\circ }$
The measured angle between tabletop longitudinal axis and couch longitudinal moving direction (B)	$(1)-1.09^{\circ }$	$(1)+0.93^{\circ }$	$(1)-2.01^{\circ }$	$(1)+2.00^{\circ }$
	$(2)-1.05^{\circ }$	$(2)+1.04^{\circ }$	$(2)-2.05^{\circ }$	$(2)+2.02^{\circ }$
	$(3)-1.03^{\circ }$	$(3)+0.97^{\circ }$	$(3)-1.98^{\circ }$	$(3)+2.03^{\circ }$
The measured angle between the plane defined by external axial lasers and imaging plane (C)	$(1)-0.90^{\circ }$	$(1)+1.02^{\circ }$	$(1)-1.99^{\circ }$	$(1)+2.06^{\circ }$
	$(2)-0.99^{\circ }$	$(2)+1.05^{\circ }$	$(2)-2.05^{\circ }$	$(2)+2.01^{\circ }$
	$(3)-0.96^{\circ }$	$(3)+1.11^{\circ }$	$(3)-2.13^{\circ }$	$(3)+1.99^{\circ }$

### B. Spatial orientations between external axial lasers, couch longitudinal moving direction, and imaging plane

**Figure 10:**
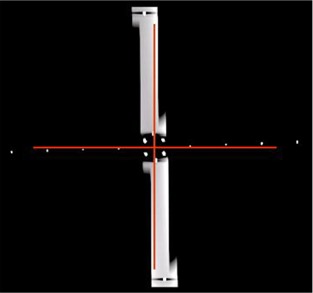
Coronal view acquired when the longitudinal arm of the jig was aligned with S line and the jig was manually moved along S line with 1.25 mm steps after acquiring each axial image. The red‐cross lines indicate the vertical and horizontal axes of the image.

The reconstructed coronal image from one of the external axial lasers orientation simulations is shown in Fig. 10. This image was acquired while we aligned the longitudinal arm of the jig with s' line and manually moved the jig along S line with 1.25 mm steps after acquiring each axial image. It is based on the simulation that there is a −2° angle (counterclockwise) between the plane defined by external axial lasers and the imaging plane, and a +2° angle (clockwise) between the couch longitudinal moving direction and the normal of imaging plane. The angular deviations were calculated by the analysis program and are tabulated on Table 4. We also have repeated the simulations with 1° orientation deviation. These tests were performed three times and the results are listed in Table 3. Again, these results have been subtracted by the inherent deviations measured by TG 66.

**Table 4:** The orientation deviations from the image showed on Fig. 7 were calculated by the MATLAB program. It is based on the situation that there is a $-2^{\circ }$ angle (counterclockwise) between the plane defined by external axial lasers and the imaging plane, and a +2° angle (clockwise) between the couch longitudinal moving direction and the normal of imaging plane

	*Expected Deviation*	*Measured Deviation*
The angle between couch longitudinal moving direction and the normal of imaging plane	$+2.00^{\circ }$	$+1.97^{\circ }$
The angle between the plane defined by external axial lasers and imaging plane	$-2.00^{\circ }$	$-2.08^{\circ }$

## III. DISCUSSION

From Table 1, we can see the “inherent” system deviations measured with both methods are all very small and similar. The considerable difference between these two methods is the time spent on performing the QA tests. In order to quantify the deviations of all spatial orientations, in TG 66, at least four tests have to be performed with multiple phantom setups. However, in the new method, we require only two CT scans with simple setup of the cross‐jig — lock to the tabletop and align with the external axial laser. The routine QA can be done more efficiently with the proposed method.

From Table 3 we can clearly see that all of the simulated misalignment orientation angles were accurately calculated by the analysis program. This validates Eq. sets (1) and (2) and this method, along with the analysis program, can be used to measure any angular deviations between the tabletop, external axial lasers, couch longitudinal moving direction, and imaging plane.

It is important to use CT markers for performing these tests. The uncertainty of the image analysis seems to be dependent on the accuracy of outlining the CT markers. Markers which produces artifact on the image result in inaccurate coordinate calculation by the program. It is recommended that we use a CT marker, which produces as few artifacts as possible.

Slice thickness is another factor that can affect the results. The smaller the slice thickness, the better the accuracy is. The slice thickness we set in this study is 1.25 mm. If we use larger thickness, for example 2.5 mm, the coordinates of CT marker can be about 1.0 mm maximum uncertainty in longitudinal direction since the diameter of CT marker is 1.5 mm. This will approximately result in about 0.14° uncertainty in our result. It is suggested that the slice thickness should be smaller than the CT marker diameter while acquiring CT images.

From the images of the simulations, we can see that potentially some of the misalignments between the components of CT simulator could result in distorted images. Unfortunately, this deformation doesn't show on individual axial image. It can be only seen on the reconstructed volumetric images, like the coronal image, as demonstrated here. Furthermore, this will result in the change in the shapes and positions of patient anatomy on CT images. This might have a severe impact on high‐precision treatment planning and evaluation.

## IV. CONCLUSIONS

We have developed a new method to determine the angular deviations of spatial orientations between tabletop, external axial lasers, couch longitudinal moving direction, and imaging plane. This procedure involves scanning the QA jig twice only, compared to the multiple scans from TG 66. The first scan is when the longitudinal arm of the jig is aligned with the longitudinal axis of the tabletop. This will detect the angular deviations between tabletop, couch longitudinal moving direction, and imaging plane. In the second scan, the transverse arm of the jig is aligned with the external axial lasers. This is going to detect the angular deviations between external axial lasers, couch longitudinal moving direction, and imaging plane. The computer program can be used to analyze the images quickly and quantitatively.

The approach proposed here is not only to simplify the spatial orientation QA procedures for CT simulator, but also can be used for quantitative comparisons. Now, we are able to obtain the exact deviation in orientation in terms of degree angles. Also, the direction of orientation deviation can be determined from the sign of the number. This will be very helpful when we need to correct the deviation.

In this study, we have tested the orientations of couch longitudinal movement and the horizontal orientations of external axial lasers. However, this concept should be applicable also to the couch vertical movement, the vertical orientations of external axial lasers, and CT gantry tilt angle measurement. We plan to design and construct a QA phantom that can verify this concept. This method can be also used to determine the spatial orientations for positron emission tomography computed tomography (PET‐CT) and magnetic resonance imaging (MRI) systems.
